# Biological factors that place women at risk for HIV: evidence from a large-scale clinical trial in Durban

**DOI:** 10.1186/s12905-016-0295-5

**Published:** 2016-03-19

**Authors:** Nathlee Samantha Abbai, Handan Wand, Gita Ramjee

**Affiliations:** HIV Prevention Research Unit, Medical Research Council, 123 Jan Hofmeyr Road, Westville, Durban, 3630 South Africa; Department of Epidemiology and Population Health, London School of Hygiene & Tropical Medicine, London, UK; The Kirby Institute, University of New South Wales, Sydney, NSW 2052 Australia

**Keywords:** HIV infection, Epithelial disruption, Abnormal vaginal discharge, Sexually transmitted infections

## Abstract

**Background:**

It is well documented that the mucosal linings of the female genital tract (FGT) usually provides a robust barrier that protects against sexually acquired infections. However, to the best of our knowledge there are limited South African studies that have investigated the association between damage to the mucosal linings and the acquisition of HIV infections. We hypothesize that in this cohort of women, a higher rate of HIV seroconversions will be observed for women who present with evidence of mucosal damage.

**Methods:**

We undertook a secondary analysis of the Methods for Improving Reproductive Health in Africa (MIRA) trial that assessed the effectiveness of the latex diaphragm and lubricant gel on HIV prevention among women. Participants underwent a physical examination which included a pelvic examination to detect the presence of mucosal abnormalities. During the physical examinations, the study clinicians examined the genitalia, cervix and vagina for signs of epithelial disruptions and abnormal vaginal discharge. The association between the various genital factors and HIV seroconversion was modeled using Cox proportional hazards regression analysis.

**Results:**

In this cohort of 1485 women that had enrolled to participate in the study, women that had presented with genital epithelial disruptions and abnormal vaginal discharge were shown to be at highest risk for HIV acquisition (Hazard Ratio (HR): 4.30, 95 % CI: 2.25, 8.22, *p* <0001, HR: 2.37, 95 % CI: 1.69, 3.33, *p* <0.001) respectively. In addition, the Kaplan Meier analysis showed that the highest number of seroconversions was observed in women that had disrupted genital epithelia (27 per 100/py, 95 % CI: 15.0, 50.7) and abnormal vaginal discharge (12 per 100/py, 95 % CI: 9.70, 16.7). Other significant factors included: genital signs and symptoms (HR: 1.67, 95 % CI: 1.07, 2.61, *p* = 0.02) and genital ulcers/sores (HR: 1.79, 95 % CI: 1.05, 3.06, *p* = 0.03).

**Conclusion:**

We have shown that damage to the mucosal epithelial lining increases a women’s risk of HIV seroconversion. Future studies that provide an in depth understanding of the mechanisms associated with the FGT and mucosal immunity will be most valuable. An understanding of all of these mechanisms will be key in directing the advancement of products most suitable for combating HIV infection in women.

**Trial registration:**

This study was registered with ClinicalTrials.gov,number NCT00121459 on the 28^th^ February 2007.

## Background

In sub-Saharan Africa (SSA), 60 % of the adults living with Human Immunodeficiency virus (HIV) infection are women [1]. It is suggested that 30–40 % of these infections occur through heterosexual transmission via the female genital tract (FGT) [2]. The FGT is divided into two main compartments; the lower reproductive tract, consisting of the vagina and ectocervix, lined by stratified squamous epithelium; and the upper reproductive tract consisting of the endocervix, endometrium and fallopian tubes, lined by a single layer of columnar epithelium [2–4]. There are numerous characteristics of FGT that have shown to increase susceptibility to HIV infection [5]. One of the characteristics included local changes in the FGT induced by infection by other microorganisms [5]. Evidence from laboratory, clinical as well as epidemiological studies have supported the hypothesis that sexually transmitted infections (STIs) facilitate the spread of HIV [6]. Sexually transmitted infections have been found to facilitate HIV transmission by rupturing protective mucosal barriers and recruiting susceptible immune cells (eg. CD4 T-helper cells, macrophages) to the site of infection [7]. Sexually transmitted infections have also been considered major causes of inflammatory cytokine up-regulation and recruitment of immune cells to the genital mucosa [8, 9]. South African studies have showed that *Chlamydia trachomatis*, *Neisseria gonorrhoeae*, *Mycoplasma genitalium* infections and elevated cervicovaginal lavage (CVL) concentrations of interleukin (IL)-1β, IL-6, IL-8 and soluble CD40L (sCD40L) are associated with increased risk of HIV acquisition [10].

Many epidemiological studies have found a strong association between HSV-2 infection and HIV infection [11]. Some studies have reported high prevalence rates in the general population as well as high risk populations. The prevalence of HSV-2 was shown to be >50 % among women and >25 % among men in Sub Saharan Africa [12]. In a more recent study by Abbai et al. [13], the prevalence of HSV-2 and HIV-1 co-infection was reported to be 41 % in women in South Africa. The increased susceptibility to HIV is most likely due to HSV-2-induced ulcerations, which ruptures the physical barrier of the genital epithelium [2]. Studies conducted using a Simian immunodeficiency virus (SIV) model has shown that damage to the mucosal barrier before SIV infection predicts disease progression to AIDS after infection, demonstrating the importance of barrier health even before infection occurs [14].

To the best of our knowledge there are limited South African studies that have investigated the association between damage to the mucosal linings and the risk for HIV acquisition. We hypothesize that in this cohort of women, a higher rate of HIV seroconversions will be observed for women who present with evidence of mucosal damage. Identifying women who present with mucosal damage could facilitate efforts that are directed towards developing products that cover the cervix and has the potential to offer safe and effective protection against HIV and other STIs. However, efforts should not be solely focused on diaphragms since diaphragms have provided no beneficial effect in preventing heterosexual HIV transmission [15].

## Methods

### Study sites

The Methods for Improving Reproductive Health in Africa (MIRA) study which assessed the effectiveness of the latex diaphragm with a lubricant gel in preventing heterosexual acquisition of HIV among women in southern Africa was conducted between 2003–2005 in South Africa (Durban) and Zimbabwe [15]. However, the provision of diaphragms and lubricant gels in addition to male condoms provided no beneficial effect [15]. This analysis is based only on participants recruited and enrolled at the Durban sites. In Durban, the study was conducted in rural Umkomaas (a peri-urban region located 60 km south of Durban) and Bothas Hill (a rural area located 40 km west of Durban).

### Study participants

Of the 3472 women screened at the Durban research sites, 1485 women were enrolled into the study. The study population has been described in greater detail elsewhere [15]. Women were recruited from family planning, well-baby and general health clinics for participation in this study. The main eligibility criteria for the study included: women aged 18 to 49 years old, sexually active (coital frequency at least four times per month on average), HIV-negative based on testing within 14 days prior to enrollment, *Chlamydia trachomatis-* and *Neisseria gonorrhoeae*-negative based on testing within 30 days prior to enrollment or, if positive, completes treatment before enrollment, have a healthy cervix as assessed by naked-eye speculum exam at enrollment, planning to live in the study area for the duration of the study, and willing and able to give informed consent. Participants provided written informed consent prior to study procedures being conducted. Participants were followed quarterly with a mean follow-up of 18 woman-months per participant. Participants had completed physical examinations at all study visits to identify any abnormalities of the cervix and to detect any cervicovaginal infections. Testing for HIV and STIs were also done at all study visits.

### Ethics

The original trial study was reviewed and approved by the University of California at San Francisco Institutional Review Board Committee on Human Research, and by the ethics review committees at all local institutions and collaborating organizations, including the Medical Research Council of Zimbabwe, the Medicines Control Authority of Zimbabwe, Biomedical Research Ethics Committee of the University of KwaZulu-Natal, Human Research Ethics Committee of the University of the Witwatersrand, and Western Institutional Review Board. This study was registered with ClinicalTrials.gov,number NCT00121459 on the 28^th^ February 2007. Approval to use any data for secondary analysis has been previously granted by the MIRA protocol team.

### Clinical data collection

At screening (baseline visit), women were interviewed by the study staff to collect information on demographics (age, marital status, religion, language spoken) and sexual behavior (number of lifetime sexual partners, age of sexual debut, sex in exchange for goods, condom use and coital frequency). Data collected on clinical history included data on treatment of STIs or reproductive tract infections, symptoms of genital ulcers, genital warts and abnormal vaginal discharge, pain in urinating, genital signs, genital irritation, painful urination and pain during intercourse. All of the above mentioned data was collected by interviewer administered questionnaires and the data recorded was based on self-report. Participants underwent a physical examination which included a pelvic examination to detect the presence of mucosal abnormalities. The external genitalia, cervix and vagina were inspected with the naked eye. During the physical examinations, the study clinicians examined the perineum/perianal, vulva and vaginal epithelium of the women for signs of epithelial disruptions. Epithelial disruption was defined as a break in the lining of the epithelium thereby exposing the subcutaneous tissue. All women that had participated in the study were compensated (monetary compensation) for their time spent at the research clinic.

### Laboratory procedures

Two HIV rapid tests were done on whole blood samples collected by venipuncture. The rapid tests used were the Determine HIV-1/2 (Abbott Laboratories, Japan) and Oraquick (OraSure Technologies, USA). Urine specimens were collected for polymerase chain reaction (PCR) testing for *Neisseria gonorrhea* (NG), *Chlamydia trachomatis* (CT) and *Trichomonas vaginalis* (TV) (Roche Pharmaceuticals, USA). Whole blood samples were also collected for the testing of syphilis and HSV-2. Testing for syphilis was conducted using the Rapid plasma regain (RPR) and *Treponema pallidum* haemagglutinin [TPHA], Randox Laboratories, Crumlin, UK). Testing for HSV-2 was conducted using a HSV2 ELISA assay (FOCUS Diagnostics, Cypress, CA, USA). The presence of bacterial vaginosis (BV) and candidiasis were based on vaginal pH and wet mount tests.

### Data analysis

The biological factors that formed part of the analysis plan included: pre-existing STIs, genital ulcer/sores, reproductive tract infections, abnormal vaginal discharge, genital signs and symptoms, genital discharge, genital irritation, genital disruption and the presence of genital warts. The date of seroconversion was estimated using the midpoint between the last negative and the first positive antibody test results within the follow-up period. Cox regression models were used to assess the associations between the risk factors and HIV seroconversion. The analysis was conducted after adjusting for age, number of lifetime sexual partners, marital and cohabitation status, age of sexual debut and education level. Kaplan-Meier survival analyses was carried out to estimate the crude HIV seroconversion rates taking into consideration the variables that were strongly associated with HIV risk such as epithelial disruptions and vaginal discharge. Statistical analysis was performed using STATA release 12.0 (College Station, Texas, TX, USA).

## Results

The baseline characteristics of the study population is described in Table 1. In our cohort of 1485 women that had enrolled to participate in the study, most of the women were younger than 25 years of age (42 %) and had experienced first sex after the age of 16 years (62 %). More than half of the women had not attained a high school qualification (72 %). Most women were unmarried (77 %), non-cohabiting (62 %) and unemployed (80 %). The coital frequency in these women was reported to be low with most women having had sex <3 times in the past week (77 %). Approximately one-third of the women reported having one lifetime sex partner (33 %) and not having used condoms in the past 3 months prior to enrollment (35 %). In addition, most women were diagnosed with HSV-2 infection (66 %) whereas fewer women were diagnosed with non-ulcerative STIs such as *N. gonorrhoeae*, *C. trachomatis*, and *T. vaginalis* (17 %). Fewer women reported experiencing abnormal vaginal discharge (18 %).

**Table 1:** Baseline characteristics of the women enrolled in the MIRA study at the Durban research sites

Variable	Total *N* (%) 1485 (100)
Age	
<25 years old	622 (42)
25–34 years old	455 (31)
>35 years old	407 (27)
Level of education	
Less than high school	1074 (72)
At least high school	411 (28)
Employment status	
Not employed	1194 (80)
Employed	291 (20)
Marital status	
Not married	1137 (77)
Married	348 (23)
Cohabitation status	
Non-cohabiting	914 (62)
Cohabiting	571 (38)
Number of life-time sexual partners	
1	492 (33)
2	464 (31)
3	262 (18)
4 +	267 (18)
Coital frequency per week	
<3	1145 (77)
>3	340 (23)
Condom use in past 3 months at enrolment	
Never	520 (35)
Sometimes	473 (32)
Always	483 (33)
Age of sexual debut	
<16 years of age	927 (62)
>16 years of age	557 (38)
Abnormal vaginal discharge	
No	1215 (82)
Yes	270 (18)
Pre-Existing STIs (*N. gonorrhoeae, C. trachomatis, T. vaginalis*)	
No	1227 (83)
Yes	258 (17)
Pre-existing HSV-2 infection	
No	502 (34)
Yes	983 (66)

The predictors of future HIV infections is described in Table 2. A history of pre-existing STIs such as *N. gonorrhoeae, C. trachomatis* and *T. vaginalis* was not significantly associated with the risk for future HIV infections (Hazard ratio (HR) 1.05, 95 % Confidence Interval (CI): 0.70, 1.57, *p* = 0.81). Similarly, being diagnosed with syphilis was not shown to be associated with incident HIV infections (HR: 0.58, 95 % CI: 0.21, 1.58, *p* = 0.29). Similar observations were made with the data obtained on reproductive tract infections such as candidiasis (*p* = 0.30) and bacterial vaginosis (*p* = 0.19). However, having a pre-existing HSV-2 infection was shown to be strongly associated with incident HIV infections (HR: 1.72, 95 % CI: 1.01, 2.94, *p* = 0.004).

**Table 2:** Predictors for future HIV-1 infections using Cox proportional hazard models for women that had participated in the MIRA study

Risk factors	Hazard ratio (95 % confidence interval)	*p*-value
Pre-existing STIs^a^	1.05 (0.70, 1.57)	0.81
Genital ulcer/sores	1.72 (1.01, 2.94)	0.04
Pain in urinating	1.01 (0.64, 1.57)	0.96
Pre-existing HSV-2 infection	1.72 (1.18, 2.51)	0.004
Pain during sex	1.22 (0.80, 1.86)	0.34
Genital irritation	1.26 (0.73, 2.20)	0.39
Genital epithelial disruption^b^	4.3 (2.20,8.21)	<0.001
Genital signs	1.62 (1.04, 2.54)	0.03
Candidiasis	1.45 (0.70, 2.96)	0.30
Abnormal vaginal discharge	2.22 (1.57, 3.12)	<0.001
Bacterial vaginosis (symptomatic)	1.36 (0.85, 2.19)	0.19
Diagnosed with *Treponema pallidum* (syphilis)	0.58 (0.21, 1.58)	0.29
Vaginal bleeding	1.39 (0.86, 2.26)	0.17
Warts	0.75 (0.10, 5.40)	0.78

^a^Neisseria gonorrhoeae, Chlamydia trachomatis, Trichomonas vaginalis

^b^Vaginal, vulva, perineum

According to the findings of the physical examinations, disruption of the genital epithelium (HR: 4.30, 95 % CI: 2.20, 8.21, *p* <0001 and abnormal vaginal discharge (HR: 2.22, 95 % CI: 1.57, 3.21, *p* = <0.001) were the factors that were the strongest predictors for HIV acquisition. Other significant factors included: genital signs (HR: 1.62, 95 % CI: 1.04, 2.54, *p* = 0.03) and the presence of genital ulcers/sores (HR: 1.72, 95 % CI: 1.01, 2.94, *p* = 0.04). However, factors such as genital irritation, pain during sex, painful urination, vaginal bleeding, and the presence of genital warts were not significantly associated with the risk for future HIV infections.

### Survival analysis

**Fig. 1:**
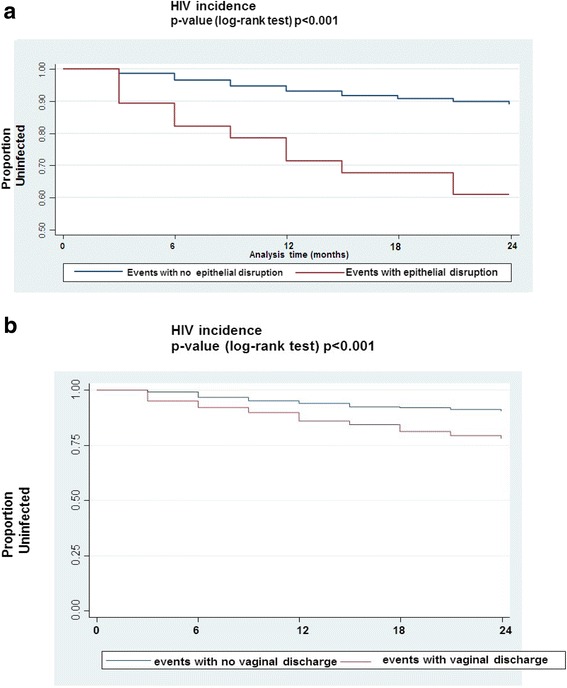
Kaplan Meier plots showing the survival rates for the individual genital factors with the crude HIV incidence rates calculated for each variable with the respective *p*-values. **a** epithelial disruption and **b** abnormal vaginal discharge

According to the Kaplan Meier survival estimates (Fig. 1), the largest number of HIV-1 seroconversions was observed with women that had symptoms of genital epithelial disruption and abnormal vaginal discharge. Approximately more than 10 % of the women that had showed signs of epithelial disruption seroconverted after being enrolled in the study for about 3 months (Fig. 1a). The overall crude HIV incidence rate associated with epithelial disruption was reported to be 27 per 100 person years (PY), (95 % CI: 15.0, 50.7). The overall crude HIV incidence rate associated with abnormal vaginal discharge was reported to be 12.7 per 100/PY, (95 % CI: 9.70, 16.7). Most women who had showed signs of abnormal vaginal discharge seroconverted after being enrolled in the study for approximately 12 months (Fig. 1b).

## Discussion

In our study, the largest number of HIV infections were reported for women who presented with signs of disruption of their genital epithelium and abnormal vaginal discharge. Our findings are in keeping with previous findings [16]. Pre-clinical studies with the SIV/macaque vaginal transmission model has shown that the normal genital tract is a substantial barrier to viral transmission and that the disruption of the epithelium is associated with enhanced HIV acquisition [16]. A reason for the enhanced susceptibility to HIV in women with epithelial disruptions has been described by Mesquita et al. [17]. According to Mesquita et al. [17], disruption of the vaginal epithelial barrier has been shown to facilitate the movement of HIV into the laminae propriae, where HIV target cells are shown to be more abundant [17]. In addition, clinical and epidemiological observation studies have also reported that the disruption of the vaginal epithelium is associated with enhanced HIV acquisition [16].

Sexually transmitted infections are known to facilitate HIV transmission by rupturing protective mucosal barriers [7]. However, in our study, we found that women who were diagnosed with STIs (*N. gonorrhoea*, *C. trachomatis* and *T. vaginalis*) were not at increased risk of HIV seroconversion. Our findings therefore differs from numerous other published studies on the association between HIV and STIs [18]. We assume that the STI treatment that was provided to our study women played an important role in protecting them from acquiring HIV. Our assumption has been confirmed by Morse et al. [19] who reported that effective treatment of curable STIs as well as HSV-2 infections have been shown to reduce the incidence of HIV infections in African populations. Additionally, observational studies also support the hypothesis that STI treatment can reduce HIV infectiousness [20].

The association between BV and HIV acquisition has been well described in a systematic review by Atashili et al. [21]. The review found that BV was consistently associated with an increased risk of HIV infection [21]. In our study, we found that women who were symptomatic for BV were at increased risk of HIV acquisition (HR 1.36), however, this result was not statistically significant. The inability to detect statistically significant effects could be attributed to the relatively small sample size of the women who had laboratory diagnoses completed for BV, resulting in reduced power.

In our study we found a large number of HIV seroconversions in women who reported abnormal vaginal discharge when compared to women who did not report any discharge. Our findings are consistent with a previous study that reported that vaginal discharge is commonly associated with women infected with HIV when compared to uninfected women [22].

In addition, in our study, women who presented with genital ulcers/sores were shown to be at risk for HIV seroconversion. Our findings are in keeping with previous reports that have shown genital ulcer diseases (GUD) to be associated with HIV infection [23]. According to Røttingen et al. [24], the effects of GUD, caused by HSV-2 on susceptibility to HIV acquisition may be greater than the effects of non-ulcerative STI symptoms (discharge, inflammation and pelvic inflammatory disease). This is consistent with our findings since women who were diagnosed with HSV-2 infections were shown to be at increased risk for HIV seroconversion. Herpes Simplex Virus type-2 has been shown to weaken the physical barrier to infection among HIV uninfected individuals as well as enhance the shedding of HIV among HIV infected individuals [6].

We acknowledge the following limitation of our study; the data presented in this study is limited to the population of women in which the research was conducted and may not be representative of women in the general population. However, the strength of the current study is that the data used for the analysis was collected from women who reside in the epicenter of the HIV epidemic in Sub-Saharan Africa and the data generated will be beneficial for developing and implementing prevention strategies that protect women from these settings from future infections.

In addition, to the best of our knowledge there have been no published reports from South African populations that have looked at the association of genital factors such as epithelial disruption and HIV seroconversion. Therefore, the findings from this study add to the growing body of knowledge regarding the biological factors that place women at risk for incident HIV infections in our current setting.

## Conclusions

In our study we have shown that damage to the mucosal epithelial lining increases a woman’s risk of HIV seroconversion. Future studies that provide an in depth understanding of the FGT and mucosal immunity will be most valuable since there are several mechanisms of transmission that are present in the FGT [25, 26]. An understanding of all of these mechanisms will be key in directing the advancement of products most suitable for combating HIV infection in women.

## References

[1] Abaasa A, Crook A, Gafos M, Anywaine Z, Levin J, Wandiembe S (2013). Long-term consistent use of a vaginal microbicide gel among HIV-1 sero-discordant couples in a phase III clinical trial (MDP 301) in rural south-west Uganda. Trials.

[2] Kaushic C, Ferreira VH, Kafka JK, Nazli A (2010). REVIEW ARTICLE: HIV infection in the female genital tract: discrete influence of the local mucosal microenvironment. Am J Reprod Immunol.

[3] Hickey DK, Patel MV, Fahey JV, Wira CR (2011). Innate and adaptive immunity at mucosal surfaces of the female reproductive tract: stratification and integration of immune protection against the transmission of sexually transmitted infections. J Reprod Immunol.

[4] Horbul JE, Schmechel SC, Miller BR, Rice SA, Southern PJ (2011). Herpes simplex virus-induced epithelial damage and susceptibility to human immunodeficiency virus type 1 infection in human cervical organ culture. PLoS One.

[5] Reis Machado J, da Silva MV, Cavellani CL, dos Reis MA, Monteiro ML, Teixeira Vde P, Miranda Corrêa RR. Mucosal Immunity in the Female Genital Tract, HIV/AIDS. BioMed Research International. 2014, 2014:20. doi:10.1155/2014/350195. 10.1155/2014/350195 PMC4181941 25313360

[6] White RG (2009). Curable sexually transmitted infection treatment interventions to prevent HIV transmission in sub-Saharan Africa. Open Infct Dis J.

[7] Kalichman SC, Pellowski J, Turner C (2011). Prevalence of sexually transmitted co-infections in people living with HIV/AIDS: systematic review with implications for using HIV treatments for prevention. Sex Transm Infect.

[8] Reddy B, Rastogi S, Das B, Salhan S, Verma S, Mittal A (2004). Cytokine expression pattern in the genital tract of chlamydia trachomatis positive infertile women—implication for T‐cell responses. Clin Exp Immunol.

[9] Sperling R, Kraus TA, Ding J, Veretennikova A, Lorde-Rollins E, Singh T (2013). Differential profiles of immune mediators and in vitro HIV infectivity between endocervical and vaginal secretions from women with chlamydia trachomatis infection: a pilot study. J Reprod Immunol.

[10] Mlisana K, Naicker N, Werner L, Roberts L, van Loggerenberg F, Baxter C (2012). Symptomatic vaginal discharge is a poor predictor of sexually transmitted infections and genital tract inflammation in high-risk women in South Africa. J Infect Dis.

[11] Glynn JR, Biraro S, Weiss HA (2009). Herpes simplex virus type 2: a key role in HIV incidence. Aids.

[12] Weiss H, Buve A, Robinson N, Van Dyck E, Kahindo M, Anagonou S (2001). The epidemiology of HSV-2 infection and its association with HIV infection in four urban African populations. Aids.

[13] Abbai NS, Wand H, Ramjee G (2015). Socio-demographic and behavioural characteristics associated with HSV-2 sero-prevalence in high risk women in KwaZulu-Natal. BMC Research Notes.

[14] Burgener A, McGowan I, Klatt NR (2015). HIV and mucosal barrier interactions: consequences for transmission and pathogenesis. Curr Opin Immunol.

[15] Padian NS, van der Straten A, Ramjee G, Chipato T, de Bruyn G, Blanchard K (2007). Diaphragm and lubricant gel for prevention of HIV acquisition in southern African women: a randomised controlled trial. Lancet.

[16] Moench TR, Chipato T, Padian NS (2001). Preventing disease by protecting the cervix: the unexplored promise of internal vaginal barrier devices. Aids.

[17] Mesquita PM, Cheshenko N, Wilson SS, Mhatre M, Guzman E, Fakioglu E (2009). Disruption of tight junctions by cellulose sulfate facilitates HIV infection: model of microbicide safety. J Infect Dis.

[18] Galvin SR, Cohen MS (2004). The role of sexually transmitted diseases in HIV transmission. Nat Rev Microbiol.

[19] Morse SA, Trees DL, Htun Y, Radebe F, Orle KA, Dangor Y (1997). Comparison of clinical diagnosis and standard laboratory and molecular methods for the diagnosis of genital ulcer disease in Lesotho: association with human immunodeficiency virus infection. J Infect Dis.

[20] Hayes R, Watson-Jones D, Celum C, van de Wijgert J, Wasserheit J. Treatment of sexually transmitted infections for HIV prevention: end of the road or new beginning? London, England: AIDS; 2010, 24(0 4). doi:10.1097/01.aids.0000390704.35642.47. 10.1097/01.aids.0000390704.35642.47 PMC3827743 21042049

[21] Atashili J, Poole C, Ndumbe PM, Adimora AA, Smith JS (2008). Bacterial vaginosis and HIV acquisition: a meta-analysis of published studies. AIDS (London, England).

[22] Greenblatt RM, Bacchetti P, Barkan S, Augenbraun M, Silver S, Delapenha R (1999). Lower genital tract infections among HIV‐infected and high‐risk uninfected women: findings of the Women's interagency HIV study (WIHS). Sex Transm Dis.

[23] Dickerson MC, Johnston J, Delea TE, White A, Andrews E (1996). The causal role for genital ulcer disease as a risk factor for transmission of human immunodeficiency virus: an application of the Bradford hill criteria. Sex Transm Dis.

[24] Røttingen J-A, Cameron DW, Garnett GP (2001). A systematic review of the epidemiologic interactions between classic sexually transmitted diseases and HIV: how much really is known?. Sex Transm Dis.

[25] Yeaman GR, Asin S, Weldon S, Demian DJ, Collins JE, Gonzalez JL (2004). Chemokine receptor expression in the human ectocervix: implications for infection by the human immunodeficiency virus‐type I. Immunology.

[26] Howell AL, Asin SN, Yeaman GR, Wira CR (2005). HIV-1 infection of the female reproductive tract. Curr HIV/AIDS Rep.

